# A Comparison of the Effects of Stochastic Resonance Therapy, Whole-Body Vibration, and Balance Training on Pain Perception and Sensorimotor Function in Patients With Chronic Nonspecific Neck Pain: Protocol for a Randomized Controlled Trial

**DOI:** 10.2196/34430

**Published:** 2022-06-24

**Authors:** Emmanuel Osinachi Igbokwe, Wolfgang Taube, Konstantin Beinert

**Affiliations:** 1 Department of Neurosciences and Movement Science Faculty of Science and Medicine University of Fribourg Fribourg Switzerland; 2 Reha Center Michaeliskarree Hof Germany; 3 Department of Physiotherapy Deutsche Hochschule fuer Gesundheit und Sport Berlin Germany

**Keywords:** neck pain, stochastic resonance therapy, whole-body vibration, cervical joint position sense, pressure pain threshold, balance training, chronic neck pain, sensorimotor function, rehabilitation, pain, therapy, chronic pain, rehabilitative technology, chronic nonspecific neck pain

## Abstract

**Background:**

Neck pain is a prevalent pathological condition, and together with low back pain, it presents as the leading cause of years lived with disability worldwide in 2015 and continues to contribute substantially to the global burden of disease.

**Objective:**

This study will investigate and compare the effects of stochastic resonance therapy (SRT), whole-body vibration (WBV), and balance training (BLT) in the management of chronic nonspecific neck pain.

**Methods:**

In total, 45 participants with chronic neck pain will be randomly allocated into SRT, WBV, and BLT groups. Pain intensity, pressure pain threshold, neck disability, and cervical joint position sense will be measured before, immediately after, and 15 minutes after the first intervention session and after 4 weeks of intervention. A follow-up postintervention measurement would be taken after 4 weeks. The SRT group will train on an SRT device (SRT Zeptor Medical plus noise, Zeptoring). The WBV group will train on a Galileo vibration device (Novotec Medical), while the BLT group will perform balance exercises. All participants shall train 3 times a week for a period of 4 weeks. Mixed ANOVA will be used to determine the main and effects of interactions within (before intervention, post intervention 1, post intervention 2, post intervention 3, and follow-up) and between (SRT, WBV, and BLT) factors on the study outcome variables.

**Results:**

Recruitment of participants started in May 2021, and as of May 2022, a total of 20 patients have been enrolled in the study. All participants are expected to have completed the trial by the end of 2022, and data analysis will commence thereafter.

**Conclusions:**

The outcome of this study will shed closer light on the effects of SRT, WBV, and BLT on pain and function in patients with chronic neck pain.

**Trial Registration:**

German Clinical Trials Register DRKS00023881; https://tinyurl.com/ycxuhj37

**International Registered Report Identifier (IRRID):**

DERR1-10.2196/34430

## Introduction

Neck pain is a prevalent pathological condition, and together with low back pain, it presents as the leading cause of years lived with disability worldwide in 2015 [1] and continues to contribute substantially to the global burden of disease. The point prevalence of neck pain from the systematic analysis of the Global Burden of Diseases, Injuries, and Risk Factors Study in 2017 is estimated at 3% to 4% per 100,000 population [2]. The recurrence and chronicity of neck pain are further estimated to be 14% to 37%, respectively [3]. In most cases of neck pain, except for cases of diagnosed myelopathy and fracture, the pathomechanism of neck pain, similar to that of low back pain, is not fully understood [3-5]. Nevertheless, a variety of sensorimotor impairments have been observed in patients with neck pain, such as increased postural instability [6] and decreased cervical joint position sense (CJPS) acuity [7,8]. These sensorimotor impairments have in common that they (1) rely largely on proprioceptive information and (2) assess egocentric body orientation in space [9].

Several interventions were proposed to counteract pain and sensorimotor dysfunction in patients with chronic neck pain. Based on a recent systematic review, motor control exercises primarily targeting the deep cervical flexor muscles, yoga, and strength training may be regarded as equally effective in reducing pain and disability in patients with chronic neck pain [10]. Apart from these rather common countermeasures, there are further promising interventions for patients with neck pain, such as balance training (BLT) or vibration. The concept of BLT as a modulator or countermeasure for neck pain has been explored for the first time by Beinert and Taube [11]; their results suggest that BLT can reduce pain intensity and ameliorate cervical joint repositioning errors [11]. In contrast to repositioning or strength tasks, the neck muscles are presumed to be unconsciously activated while the individual tries to maintain body equilibrium during BLT [11]. Furthermore, BLT was shown to improve γ-aminobutyric acid–mediated (GABAergic) intracortical inhibition [12,13]. Appropriate inhibitory control is needed to suppress the perception of pain [14], and, so far, unpublished results from our laboratory demonstrate increased intracortical inhibition co-occurring with reduced pain perception in a patient experiencing pain after the administration of BLT.

Another intervention that reduces pain and improves CJPS in patients with neck pain is locally applied vibration [15,16]. Vibration can be applied locally on specific muscles or tendons or as whole-body vibration (WBV) [17]. Both types of vibration stimulate proprioceptors; for example, muscle spindles [18,19]. This is of interest because the deep layers of cervical flexor and extensor muscles display a large number of muscle spindles and might contribute with their connections to the vestibular system [20,21] to the egocentric body orientation in space [20]. The mechanism by which vibration activates muscles is described as the “tonic vibration reflex” [19,22]. It is believed that the vibration stimuli excite the muscle spindles, which, in turn send signals to the spinal cord where the polysynaptic reflex system is activated, consequently causing muscle contractions [17,22,23].

Clinically, applying local muscle vibration in patients with neck pain reduced neck pain and improved CJPS [15,16]. Thus far, the application of WBV in patients with chronic neck pain has not been studied, but research shows that neck muscles are stimulated during WBV training [24]. Thus, WBV may be a potential treatment candidate that can reduce pain and improve sensorimotor functions in patients with neck pain. In other studies that did not include patients with neck pain, potential therapeutic benefits of WBV have been reported to include positive effects in patients with cerebral palsy [25], potential improvement of CJPS in people with forward head posture [17], and reduction of pain in patients with chronic low back pain [25,26].

Stochastic resonance therapy (SRT) is a form of whole-body vibration therapy that uses randomized stimuli that are amplified with the help of noise [27]. The vibration output from SRT is different from that of a regular WBV in that it is random and unpredictable. Thus, the patient is constantly challenged to adapt to the multidimensional perturbations from the device’s standing platform [28,29]. Previous studies have suggested potential therapeutic benefits of the SRT [28,30-32]. For example, Kaut et al [31] reported increased postural stability in patients with Parkinson disease after administration of SRT. SRT is also known to reduce musculoskeletal pains [32,33]. Furthermore, a recent systematic review and meta-analysis indicated improvements in postural control and balance performance in healthy young adults, older adults, and people with lower extremity injuries after stochastic stimulation [34].

Until now, the effects of SRT and WBV in cervical pain are not known. Thus, this study aims to answer the research question which treatment among BLT, WBV, and SRT has the greatest effect in reducing pain intensity, the pressure pain threshold (PPT), and neck disability and in improving CJPS acuity in patients with chronic neck pain.

## Methods

### Trial Design

The trial uses a single-blind randomized controlled design and conforms to the Standard Protocol Items: Recommendations for Interventional Trials guidelines [35].

### Study Setting and Participants

The study will be conducted at the Reha Center Michaeliskarree GmbH (an outpatient rehabilitation center) in Hof, Bavaria, Germany. Participants will be recruited through advertisement posters placed at the rehabilitation center and other health facilities in the neighborhood. We will recruit a total of 45 participants for the study, who are allocated to 3 arms of 15 participants per group: SRT, WBV, and BLT. We plan to recruit 2 additional patients per group to be sure that our design will not be underpowered in case of an approximately 15% dropout.

**Figure 1:**
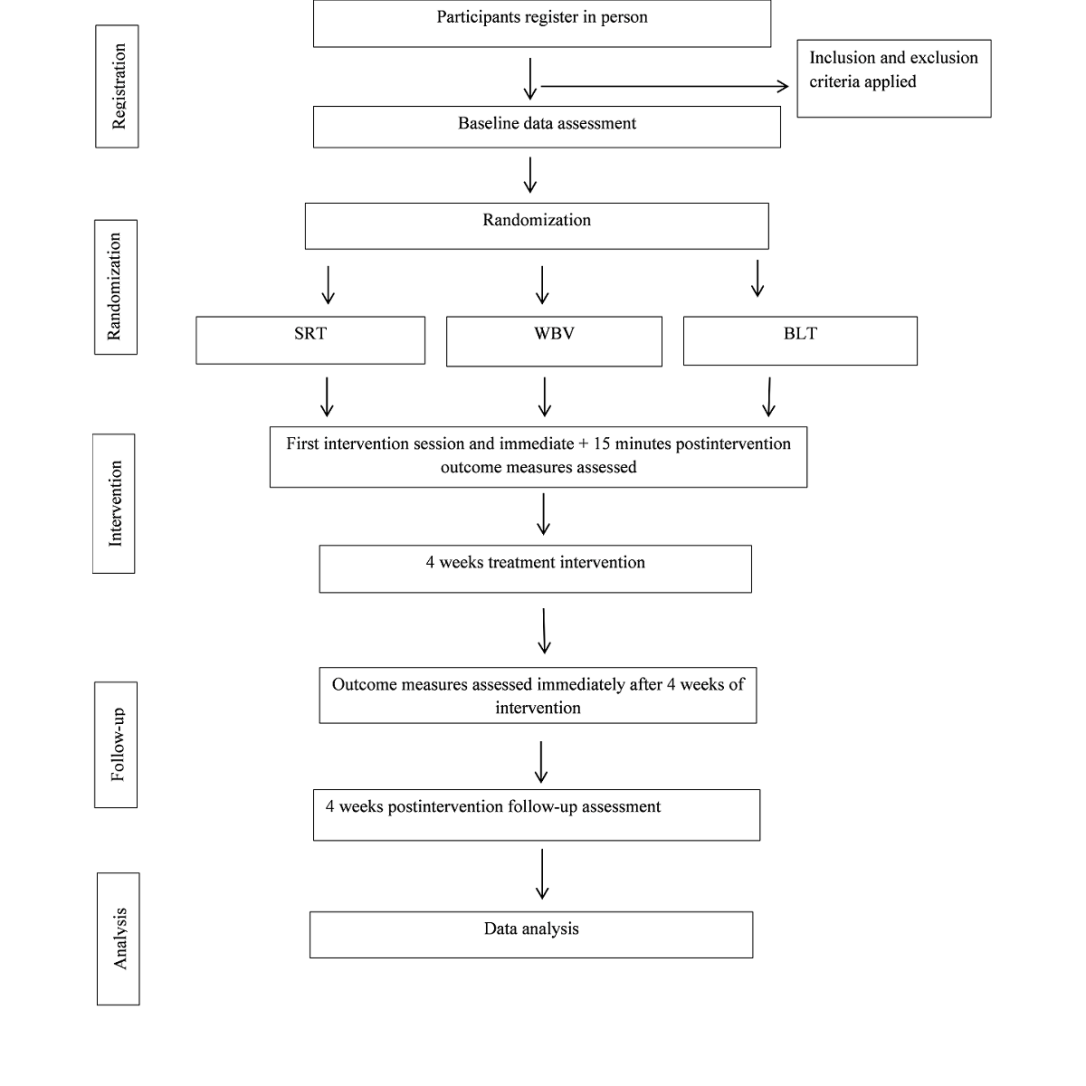
Flowchart of the study protocol. BLT: balance training, SRT: stochastic resonance therapy, WBV: whole-body vibration.

The progression of the trial is summarized in Figure 1. Participants will be included in the study if they have grade 1 neck pain without daily interference and grade 2 neck pain, which interferes with daily activities [36] for 3 months or longer, are between the ages of 18 and 65 years, and sign a written informed consent form. The following exclusion criteria are applied: the presence of potential contraindications to vibrations (eg, pregnancy and fresh medical procedures), uncontrolled high blood pressure or low blood pressure, kidney stones, glaucoma, acute thrombosis, advanced osteoporosis, presence of neurological conditions, epilepsy, rheumatoid arthritis, acute hernia, schizophrenia, pacemaker, aneurysm, heart arrhythmias and tumors or metastases, and uncontrolled diabetes. Furthermore, patients with the following pre-existing conditions shall be excluded: vertigo, dizziness, diabetic disorders, vertebrobasilar insufficiency, limiting pain, difficulties understanding instructions, difficulties adopting the required posture on the devices, whiplash-associated disorder, and traumatic injury to the neck.

### Interventions

All 3 groups will train 3 times per week for 4 weeks. The SRT group will train on an SRT device (SRT Zeptor Medical-plus noise, Zeptoring), while the WBV group will train on Galileo Sport (Novotec Medical). The BLT group will perform balance exercises on different unstable devices. All participants will be instructed not to seek any other neck-specific therapy except in case of an emergency, and such events should be documented and reported to the researchers.

### Training Protocol for SRT and WBV Groups

The participants are instructed on how to use the devices. In the first 3 visits (first week), the participants will train while standing on both legs on the device surface with slightly bent knees at approximately 30°. As from the second week of intervention, the training will progress to include more challenging activities on the device (see Table 1).

The participants are instructed to place one leg at the front and the other one behind, similar to a semitandem stance. Following this, participants are invited to alternately raise one leg and stand on the other leg for 30 seconds first and later transfer a ball from one hand to the other. Furthermore, the trainer throws a ball to the participants, which they catch and throw back to the trainer. Finally, they are asked to try to close their eyes while standing on one leg. The progression of the challenges is implemented for each participant on an individual basis.

**Table 1:** Description of the intervention’s progression.

Exercise levels and details				Targeted repetitions
**Balance training [37]**				
	**Level 1 (static balance)**			
		Static stance (wobbly and spinning surfaces with eyes opened or closed)	30 s hold × 4 repetitions	
		Single leg stance (firm, soft, wobbly, or spinning surfaces with eyes opened or closed)	30 s hold × 6 repetitions	
		Tandem stance (firm, soft, wobbly, or spinning surfaces with eyes opened or closed)	30 s hold × 6 repetitions	
	**Level 2 (dynamic balance with mental tasks)**			
		Throwing or catching a ball while tandem stance (on firm, soft, wobbly, or spinning surfaces, counting backward in multiples of 7, or reciting the alphabet backward from Z to A)	30 s hold × 6 repetitions	
		Throwing or catching a ball while single leg standing (on firm, soft, wobbly, or spinning surfaces, counting backward in multiples of 7, or reciting the alphabet backward from Z to A)	30 s hold × 6 repetitions	
		Tandem walking (forward or backward, on a soft surface, with eyes opened or closed, counting backward in multiples of 7, or reciting the alphabet backward from Z to A)	10 repetitions	
**Stochastic resonance therapy (2-6 Hz, 3 mm)**				
	**Level 1 (static balance)**			
		Two-legged stance	60 s × 8 repetitions	
		Tandem stance	60 s × 8 repetitions	
		Single leg stance (eyes opened or closed)	30 s × 16 repetitions	
	**Level 2 (dynamic balance)**			
		Throwing or catching a ball while standing on a single leg	30 s × 16 repetitions	
		Transferring a ball from one hand to the other while standing on a single leg	30 s × 16 repetitions	
	**Level 3 (dynamic balance with mental tasks)**			
		Throwing or catching a ball while standing on a single leg (counting backward in multiples of 7 or reciting the alphabet backward from Z to A)	30 s × 16 repetitions	
**Whole-body vibration (5 Hz, 4 mm)**				
	**Level 1 (static balance)**			
		Two-legged stance	8 min × 1 repetition	
		Tandem stance	8 min × 1 repetition	
		Single leg stance (eyes opened or closed)	30 s × 16 repetitions	
	**Level 2 (dynamic balance)**			
		Throwing or catching a ball while standing on a single leg	30 s × 16 repetitions	
		Transferring a ball from one hand to the other while standing on a single leg	30 s × 16 repetitions	
	**Level 3 (dynamic balance with mental tasks)**			
		Throwing or catching a ball while standing on a single leg (counting backward in multiples of 7 or reciting the alphabet backward from Z to A)	30 s × 16 repetitions	

### Training Parameters for SRT and Galileo Vibration Devices

#### Stochastic Resonance Therapy (SRT)

The “medium trim” of the program “chronic head, neck and back pain by muscle imbalance” will be selected on the SRT device. The vibration frequency of this program ranges between 2 Hz and 6 Hz at an amplitude of 3 mm. The program comprises 8 series of vibration bouts, each lasting 60 seconds with a 60-second break between bouts. The last bout is for cooling down.

#### Galileo Vibration

The Galileo device elicits a side alternating vibration at a 4-mm amplitude. Its frequency will be adjusted to 5 Hz to ensure that the settings of both the Galileo and SRT devices are similar. The participants train on the device for a total duration of 15 minutes (including 8 minutes of active training and a 7-minute break).

### Training Protocol for the BLT Group

The BLT group will perform 15 minutes of balance exercise training (8 minutes active training and a 7-minute break) 3 times per week for 4 weeks. Each training session shall comprise different balance exercises, and the difficulty of the exercises will be progressively adjusted throughout the training (see Table 1). Participants will have to stand on wobbling boards, spinning tops, soft mats, and other devices with a reduced base of support. For exercises performed on one leg, one leg is trained first for 30 seconds and the other leg afterward for the same duration. Furthermore, task difficulty will be progressively increased (smaller base of support, standing with eyes closed, catching a ball, counting backward in steps of 7, etc) for each participant in an individual and adapted manner. There will be a 30-second rest period after every set of exercises.

### Outcome Measures

#### Overview

Pre- and postintervention measurements and outcome data shall be assessed by EOI. EOI is a sport scientist with a cumulative work experience of 6 years as a physical education teacher and sports therapist. EOI is trained in using the underlisted assessment instruments and procedures.

Pain intensity, PPT, neck disability, and CJPS are measured before, immediately after, and 15 minutes after the first intervention session and after 4 weeks of the intervention. A follow-up postintervention measurement will be taken after 4 weeks.

#### Pain Intensity

Pain intensity will be assessed using the numeric rating scale (NRS) for pain. The NRS is a reliable (0.67) and valid (0.67) instrument for measuring pain in patients with neck pain without upper extremity symptoms [38]. The NRS has also been shown to be sensitive to intervention-induced changes [38].

#### Cervical Joint Position Sense (CJPS)

The participants will wear on their head a cervical goniometer (CROM, Performance Attainment Associates) attached with a laser pointer (P2, NOBO) and will be required to sit straight and comfortably without resting their back on a chair placed 90 cm away from the target. The target is a board that can be adjusted so that the center of the board is at eye level for each participant when seated and looking straight ahead. This initial head position is defined as the neutral head position (NHP) and will be marked on the target screen. From this NHP, the blindfolded patients are instructed to rotate their head to the side and then to turn their head back to the NHP and provide a verbal signal by saying “now” when they think they have returned to the NHP with the laser pointer. The point at which they made the stop signal is marked, and the distance between this point and the NHP is measured in centimeters. This procedure will be repeated 8 times for right as well as left head rotations. A systematic review of the literature by de Vries et al [8] and colleagues demonstrated that CJPS tests are efficient in identifying CJPS acuity errors in patients with neck pain when at least 6 repetitions are performed. The CJPS test has fair to high test-retest reliability (intraclass correlation coefficient=0.39-0.78) [39] and good discriminative, convergent, and divergent validity scores [40].

#### Neck Disability

The German version of the Neck Pain and Disability Scale (NPAD-d) will be used to assess the functional capacity of the patients. The NPAD-d has an excellent reliability and validity score (Cronbach α=.94) [41].

#### Pressure Pain Threshold (PPT)

The PPT will be measured using a pressure algometer (Wagner Instruments). Standing behind the seated participant, the assessor will incrementally apply pressure on the trigger point of the levator scapulae muscle at the angulus superior of the scapula until the participant calls out “now” to indicate the onset of painful pressure. The assessor stops immediately and records the reading on the algometer at which point the participant started to feel pressure pain. To enhance the reliability of the test, 3 repetitions are carried out on the left and right sides. The PPT test has high interrater and test-retest reliability (intraclass correlation coefficient=0.75-0.95) as reported in a previous study [42].

### Randomization and Allocation of Concealment

The participants will be randomly allocated to the SRT, WBV, or BLT group. Randomization will be performed electronically using the QuickCalc software (GraphPad Software) and sealed in a brown nontransparent envelope. A physiotherapist who is not involved in the study will allocate the patients into groups. The sequencing and allocation will be concealed from the participants and other members of the research team. Furthermore, the analyst is blinded to the actual treatment of participants. All the actors involved will be unblinded after the final measurement from the last patient.

### Sample Size

The total sample and subgroup size were determined by conducting an a priori power analysis using the G-Power software (version 3.1.9.7; G-Power) by applying repeated measures ANOVA and selecting within- and between-group interactions with an effect size *f* set at 0.4 (large effect size), α of .05, and β of .90. Estimation of large effect sizes is based on previous results obtained from among patients with neck pain and on the therapy duration and frequency.

### Statistical Analysis

Statistical analyses will be performed using SPSS (version 24; SPSS Inc). Descriptive statistics will be used to report the study population’s demography and characteristics. The Gaussian distribution of data will be assessed using the Shapiro-Wilk test. Thereafter, mixed ANOVA will be used to determine the main and interaction effects of within (before intervention, after intervention 1, after intervention 2, after intervention 3, and follow-up) and between (SRT, WBV, and BLT) factors on the study outcome variables. Mean (SD) values, effect sizes, and 95% CIs are reported.

### Ethics Approval, Study Registration, and Consent to Participate

The study will be conducted in accordance with the tenets of the Declaration of Helsinki. Ethical approval was obtained from the ethics committee of the Deutsche Hochschule für Gesundheit und Sport (DHGS-EK-2021-001), and the study was registered with the German Clinical Trials Register (DRKS00023881) and in the World Health Organization’s International Clinical Trials Registry Platform (U1111-1262-6569). Participants will be required to sign the informed consent form and return it during recruitment to the same physiotherapist who informs them of their study group allocation.

### Protocol Amendments

Any major amendment to the study protocol will be communicated to the responsible ethics committee and the trial registration body.

### Confidentiality and Access to Data

Data are anonymized by asking the participants during recruitment to generate their research code by combining the last 2 letters of their mothers’ first name, the last 2 letters of their fathers’ first name, and their birth date. For example, if the first name of a participant’s mother is Helga, the first name of the father is Herbert, and patient’s date of birth is December 5, 1992, the participant’s code is GART12. Data will be safely stored in a lockable cupboard and can only be accessed by the researchers.

### Safety and Data Monitoring

Participants are insured by the existing insurance policy at Reha Center Michaeliskarree. During the trial, participants will be continuously monitored for possible occurrence of any side effects of the treatments. Most side effects as reported from previous studies, and as observed by the investigators, are minor and disappear shortly afterward. In the event of an established more serious side effect, the patient will be immediately referred to the hospital. All documented side effects will be reported in a separate publication. The lead investigator and research team will monitor the safety and scientific integrity of the trial.

## Results

Recruitment of participants started in May 2021. In total, 20 patients have been enrolled as of May 2022. All participants are expected to have completed the trial by the end of 2022, and analysis of data will commence thereafter.

## Discussion

### Expected Findings

We hypothesize that all 3 interventions will reduce neck pain and improve the sensorimotor function of the cervical spine. Furthermore, we hypothesize that SRT elicits larger effects than WBV owing to the random and multidimensional nature of the former, thus being a more challenging application of the vibration stimuli. Finally, we hypothesize that BLT elicits the most beneficial effects as it incorporates the coordinatively most challenging tasks and is known to upregulate the inhibitory GABAergic system in the brain, which is also needed to suppress the perception of pain.

Manufacturers of vibration devices have touted their benefits for muscle activation, bone strengthening, and treatment of several health conditions. Indeed, some of these claims are supported by research, as suggested by the latest up-to-date reviews of the literature and meta-analysis [43,44]. However, to the best of our knowledge, this would be the first study to investigate the treatment effects of SRT and WBV in patients with chronic nonspecific neck pain and to compare these effects with BLT. The results from this study will contribute to our understanding of the role of WBV (stochastic and nonstochastic) and BLT in the management of chronic neck pain. It is hoped that this study spurs further research in the application of SRT, WBV, and BLT as treatment options for neck pain. Future studies should further explore how the human neck region responds to WBV stimuli and if this response is correlated with pain reduction and sensorimotor function improvement.

### Limitations

A potential limitation of the study is the strict exclusion criteria, which were set to eliminate the possible risk exposure of patients with pre-existing contraindications for WBV. As a result, some patients with neck pain who could have benefited from the treatments were excluded.

To minimize bias, analysts were blinded, but owing to the work setting at the trial center, the data assessor could not be blinded. To reduce potential assessor bias, very strict measurement criteria were applied.

### Dissemination Policy

The results will be communicated to the participants and shared publicly through publication in peer-reviewed journals and at international scientific conferences.

### Conclusions

The outcome of this study will particularly elucidate the effects of SRT, WBV, and BLT on pain and function in patients with chronic neck pain.

## Abbreviations

BLT: balance training
CJPS: cervical joint position sense
GABAergic: γ-aminobutyric acid–mediated
NHP: neutral head position
NPAD-d: German version of the Neck Pain and Disability Scale
NRS: numeric rating scale
PPT: pressure pain threshold
SRT: stochastic resonance therapy
WBV: whole-body vibration

## Data Availability

Data sets are not included in this protocol but may be released upon a reasonable request to the corresponding author.
